# Translational Control of Host Gene Expression by a Cys-Motif Protein Encoded in a Bracovirus

**DOI:** 10.1371/journal.pone.0161661

**Published:** 2016-09-06

**Authors:** Eunseong Kim, Yonggyun Kim

**Affiliations:** Department of Bioresource Sciences, Andong National University, Andong 36729, Republic of Korea; Institute of Plant Physiology and Ecology Shanghai Institutes for Biological Sciences, CHINA

## Abstract

Translational control is a strategy that various viruses use to manipulate their hosts to suppress acute antiviral response. Polydnaviruses, a group of insect double-stranded DNA viruses symbiotic to some endoparasitoid wasps, are divided into two genera: ichnovirus (IV) and bracovirus (BV). In IV, some Cys-motif genes are known as host translation-inhibitory factors (HTIF). The genome of endoparasitoid wasp *Cotesia plutellae* contains a Cys-motif gene (*Cp-TSP13*) homologous to an HTIF known as teratocyte-secretory protein 14 (TSP14) of *Microplitis croceipes*. Cp-TSP13 consists of 129 amino acid residues with a predicted molecular weight of 13.987 kDa and pI value of 7.928. Genomic DNA region encoding its open reading frame has three introns. Cp-TSP13 possesses six conserved cysteine residues as other Cys-motif genes functioning as HTIF. *Cp-TSP13* was expressed in *Plutella xylostella* larvae parasitized by *C*. *plutellae*. C. plutellae bracovirus (CpBV) was purified and injected into non-parasitized *P*. *xylostella* that expressed *Cp-TSP13*. *Cp-TSP13* was cloned into a eukaryotic expression vector and used to infect *Sf*9 cells to transiently express *Cp-TSP13*. The synthesized Cp-TSP13 protein was detected in culture broth. An overlaying experiment showed that the purified Cp-TSP13 entered hemocytes. It was localized in the cytosol. Recombinant Cp-TSP13 significantly inhibited protein synthesis of secretory proteins when it was added to *in vitro* cultured fat body. In addition, the recombinant Cp-TSP13 directly inhibited the translation of fat body mRNAs in *in vitro* translation assay using rabbit reticulocyte lysate. Moreover, the recombinant Cp-TSP13 significantly suppressed cellular immune responses by inhibiting hemocyte-spreading behavior. It also exhibited significant insecticidal activities by both injection and feeding routes. These results indicate that Cp-TSP13 is a viral HTIF.

## Introduction

Host regulation by endoparasitoids is necessary for successful parasitism [1,2]. These koinobionts whose hosts continue to develop as the endoparasitoids grow have to defend host immune responses and compete with growing tissues of host under restricted nutrient source. To regulate host physiological processes, several parasitic factors have been identified from some endoparasitoid wasps of superfamily Ichneumonoidea. Proteinaceous parasitic factors have been identified from either oviduct lumen or venom gland of wasps [3,4]. At early stage of parasitism, these protein factors are known to be involved in suppressing immune responses or facilitating the action of other parasitic factors [5–7]. Polydnaviral parasitic factors have been reported in some braconid or ichneumonid wasps, in which viral genes are expressed to suppress host immune response and alter host development [8]. Teratocytes are derived from extra-embryonic membrane during egg development of wasp. They play crucial roles in host immunosuppression and nutrition for wasp development during late stage of parasitism [9,10].

Of these parasitic factors, polydnaviruses (PDVs) are considered as the main host regulators with many numbers and kinds of genes encoded by them [11,12]. PDVs are a group of insect double-stranded DNA viruses. They are symbiotically associated with some braconid or ichneumonid wasps [13]. PDVs are classified into viral family of polydnaviridae based on their symbiotic wasp host families [14]. They consist of two genera: Ichnovirus (IV) and Bracovirus (BV). However, IV and BV are likely to be independently originated but have undergone a convergent evolution, thus exhibiting similar parasitic life style [14]. PDV genomes are fragmented and located on wasp host chromosome(s) [15]. Whole genome analyses of host parasitoid wasps along with ovarian transcriptomic and proteomic analyses unanimously support that BVs are originated from a common nudiviral ancestor at around 100 million years ago [16–18]. The origins of IVs are currently unknown. Proviral PDV genes are grouped into non-encapsidated (NEG) and encapsidated (EG) genomes [16,19]. NEG is presumed to be functional during viral replication by facilitating EG replication and excision as well as by producing viral coat proteins [20]. EG is multiplied and circularized to form viral particles [21]. The resulting PDV virions do not possess NEG. They fail to undergo further replication in virus-infected cells. Viral replication occurs during late pupal stage in the ovarian cells at calyx region between ovary and lateral oviduct [22,23]. During parasitization, viral particles are delivered to hemocoel of the parasitized lepidopteran host along with parasitoid egg. They can enter target cells to express their encoded genes [24,25]. Expression of PDV genes can lead to host regulation such as immunosuppression, delayed larval development, and restricted nutrients for wasp development [26–28].

Endoparasitoid wasp *Cotesia plutellae* can parasitize young larvae of the diamondback moth, *Plutella xylostella* [29]. Parasitized larvae exhibit significant immunosuppression and developmental alteration [30,31]. More than 70% of major nutrients of *P*. *xylostella* larvae can be deprived by the parasitism of *C*. *plutellae* [32]. All parasitic factors described above are involved in host regulation [33]. Especially, EG of *C*. *plutellae* bracovirus (CpBV) encodes 157 genes classified into PDV canonical gene families, *Cotesia*-specific gene families, and several hypothetical genes [34]. The most abundant family is protein-tyrosine phosphatase (PTP) that plays crucial roles in suppressing hemocyte behavior and delaying host larval development [31,35]. Early expressed gene family (EP1-like) is only specific to *Cotesia* spp. [36]. It is cytotoxic to hemocytes of *P*. *xylostella* [37]. A coiled-coil protein called CrV1 is also specific to *Cotesia* spp. It can prevent hemocyte-spreading behavior by interrupting α-tubulin-bundling via sequestering glucose-6-phosphate dehydrogenase [38].

Host translation-inhibitory factor (HTIF) was originally proposed from IV in host-parasitoid association of *Heliothis virescens* and *Campoletis sonorensis* [39]. Later, two Cys-motif IV genes VHv1.4 and VHv1.1 were identified as HTIFs [40]. Another Cys-motif gene TSP14 was identified from teratocytes of *Microplitis croceipes*. It can inhibit host mRNA translation [41]. The two Cys-motif HTIFs are not homologous in amino acid sequences. However, they share a characteristic knot-forming disulfide bonds due to six conserved cysteine residues [42]. The molecular actions of these HTIFs are not well understood. Two homologous proteins (CpBV15α and CpBV15β) encoded in CpBV are similar to eukaryotic translation initiation factors (elFs) identified as HTIFs of BVs [43,44]. CpBV15α specifically inhibits the translation of host mRNAs by hindering elF5, thus preventing the formation of 80S ribosome complex [45]. CpBV15β sequesters eIF4A to prevent a scanning process of a cap-binding protein at 5’-untranslated region of target mRNAs [46]. However, Cys-motif genes playing a role in HTIF have not been identified in BVs.

This study identified a new HTIF encoded in CpBV called *Cp-TSP13*. It shared high sequence homology with TSP14 encoded in teratocytes of *M*. *croceipes*. *Cp-TSP13* was expressed in all stages of parasitism. Its recombinant protein inhibited host mRNA translation and cellular immunity. Furthermore, it possessed potent insecticidal activity after oral administration.

## Results

### *Cp-TSP13* is a Cys-motif gene

**Fig 1:**
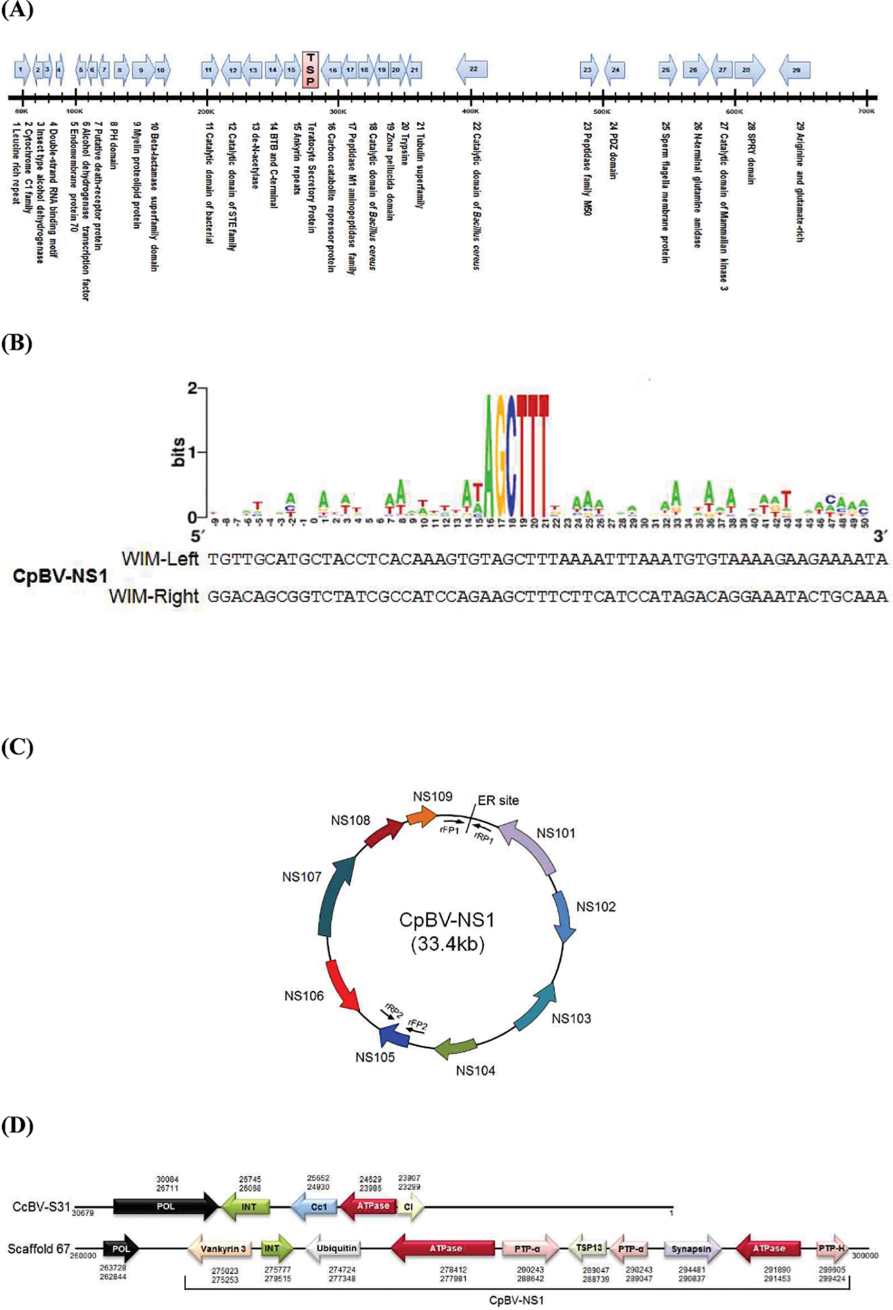
A new viral segment (CpBV-NS1) of CpBV in scaffold 67 of *C*. *plutellae* genome. (A) 27 putative open reading frames (ORFs) in scaffold 67 (751,295 bp). TSP represents teratocyte-secretory protein. Arrows indicate translation directions of the ORFs. (B) Conserved sequences of WIM (wasp integration motif) surrounding CpBV-NS1. A conserved PDV excision site sequence obtained from previous related PDVs (Burke *et al*., 2015) was used to locate the sites in different CpBV segments (NCBI accession number): S2 (DQ075354), S3 (DQ075355), S4 (DQ075356), S5 (DQ075357), S8 (DQ075358), S9 (DQ075359), S10 (EF067319), S11 (DQ075360), S14 (EF067320), S16 (EF067321), S21 (EF067322), S22 (EF067323), S27 (DQ067324), S28 (AY651829), S30 (AY651828), S33 (AY651830), S35 (EF067325), S36 (EF067326), S37 (EF067327), S38 (EF067328), S41 (EF067329), S48 (EF067330), S50 (EF067331), and S51 (EF067332). Multiple alignment of these terminal repeats of CpBV segments was performed using DNAstar program (Version 5.02, DNAstar Inc., Madison, WI, USA). Sequence logos based on the alignment were created with WebLoGo Sequence Logo Generator (http://weblogo.berkeley.edu/logo.cgi). WIM-left and WIM-right of CpBV-NS1 are denoted below sequence logos. (C) A circular map of CpBV-NS1. Ten ORFs correspond to ORF30-ORF39 of scaffold 67 of *C*. *plutellae* genome (S1 Table). ER site represents excision and rejoining site. Arrows indicate translation directions of nine ORFs encoded in CpBV-NS1. Two PCR primers (rFP1 and rRP1) were used to confirm the ER site. In the PCR assay, rFP2 and rRP2 were used as control PCR primers to indicate the existence of segment DNA on *C*. *plutellae* genome. Primer sequences are listed in Table 2. (D) Gene architecture similarity between CpBV-NS1 and CcBV-S31 (a viral segment of *C*. *congregata* bracovirus). Numbers above or below each ORF indicate their locations on CpBV-S31 or scaffold 67 of *C*. *plutellae*, respectively. Gene architecture of gene products: ‘POL’ for DNA polymerase B, ‘Cc1’ for coil-coiled protein 1, ‘Cl’ for capsid-like protein, ‘INR’ for integrase, ‘PTP’ for protein tyrosine phosphatase, and ‘Ubiquitin’ for E3 ubiquitin-protein ligase E3D.

Transcriptomic analysis of venom gland of *C*. *plutellae* identified a Cys-motif gene similar to TSP14 of *M*. *croceipes*. Interrogation with this transcript sequence to *C*. *plutellae* genome (SRX057734) selected a corresponding Cys-motif gene (TSP, Fig 1A). TSP is surrounded by a PDV canonical integration site called WIM (wasp integration motif [47]) in scaffold 67 (Fig 1B, S1 Table). This segment was named CpBV-NS1. A gene-finding program predicted 10 ORFs (Table 1, Fig 1C) in this segment. Some of these ORFs such as ATPase and integrase were highly similar to those of CcBV31 (Fig 1D). CpBV-NS1 also shared similar gene architecture with CcBV31 with respect to ORF arrangement within or nearby the segment.

**Table 1:** A new CpBV proviral segment (CpBV-NS1, 33.4 kb) containing a Cys-motif gene called teratocyte-secretory protein (TSP) and its encoding gene prediction.

ORF^1^	Location	AA (residues)	Predicted gene (species)	GenBank accession number	E-value
CpBV-NS101	275023←275253	416	Vankyrin 3 (*Microplitis demolitor* bracovirus)	XM_008555164.1	3.00E-41
CpBV-NS102	275777→279515	74	Integrase core protein(*Cotesia congregata* bracovirus)	KP706800.1	0.14
CpBV-NS103	276724←277347	360	E3 ubiquitin-protein ligase E3D(*Microplitis demolitor*)	XM_008545734.1	6.00E-16
CpBV-NS104	277981←278412	296	ATPase (*Cotesia congregata* bracovirus)	AJ632329.1	5E-45
CpBV-NS105	288642→290243	494	PTP-α (*Cotesia sesamiae* bracovirus)	CP007235.1	1.00E-94
CpBV-NS106	288739←289047	102	TSP13(*Microplitis demolitor*)	JX399877.1	1E-08
CpBV-NS107	289047←290243	117	PTP-α(*Cotesia congregata* bracovirus)	DQ839630.1	2.00E-35
CpBV-NS108	290837→294481	542	Synapsin(*Atta cephalotes*)	XM_012208289.1	3E-41
CpBV-NS109	291453→ 291890	37	ATPase (*Cotesia congregata* bracovirus)	AJ632317.1	2.30E+00
CpBV-NS110	299424→299605	182	PTP-H(*Cotesia congregata* bracovirus)	AJ640092.1	6E-111

^1^ Open reading frame (ORF) in a new segment (NS) of *Cotesia plutellae* bracovirus (CpBV)

**Fig 2:**
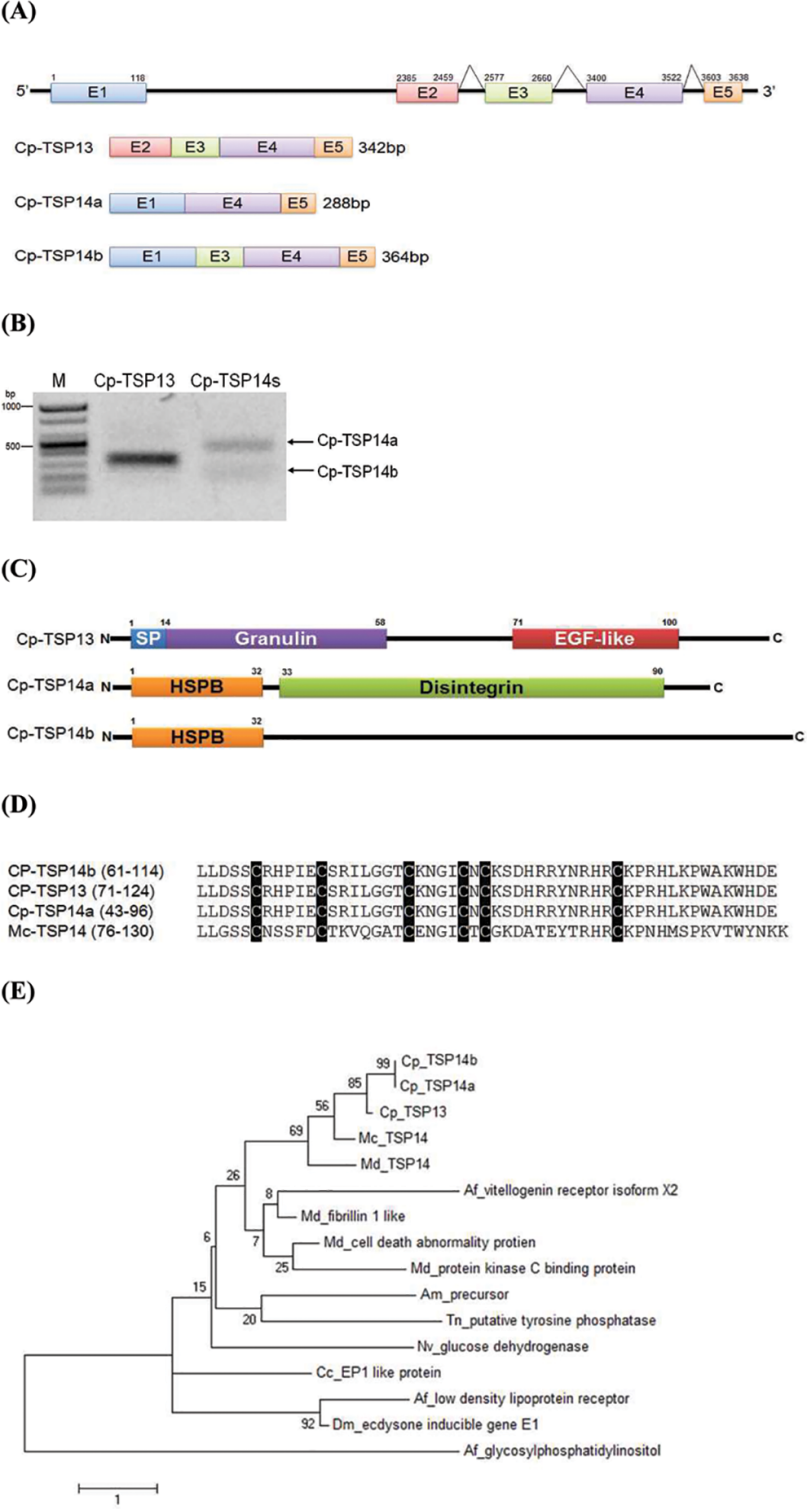
Three Cys-motif genes of *C*. *plutellae*: *Cp-TSP13*, *Cp-TSP14a*, and *Cp-TSP14b*. (A) Teratocyte-secretory protein (TSP) gene structure encoded in scaffold 67 of *C*. *plutellae*. Five exons (E1-E5) and three introns joining E2-E3 or E3-E4 or E4-E5 are denoted. Three different gene products are predicted from alternatively joining exons. (B) The three gene products were visualized by RT-PCR, in which two different forward primers at E1 and E2 were designed and a common reverse primer was designed at E5. RT-PCR of Cp-TSP13 used E2 and E5 primers, while RT-PCR of Cp-TSP14 used E1 and E5 primers. (C) Domain structure of three gene products: ‘SP’ for signal peptide, ‘Granulin’ for a family of secreted and glycosylated peptides cleaved from progranulin, ‘EGF-like’ for epidermal growth factor-like, ‘HSPB’ for heat shock protein binding motif, and ‘Disintegrin’ for antagonist against integrin. (D) A conserved nature of six cysteine residues of the three gene products with respect to known Cys-motif gene Mc-TSP14. Numbers in the parentheses indicate the locations of amino acid residues. (E) A phylogenetic analysis of Cp-TSP genes with related genes was performed with Neighbor-joining method using MEGA 5 [78] and Clustal W program of DNASTAR (Version 5.01). Bootstrapping values were obtained with 1,000 repetitions. All sequences were obtained with GenBank accession numbers: XP_001747551.1 for *Microplitis croceipes* TSP14 (McTSP14), AAN17943.1 for *Microplitis demolitor* TSP14 (MdTSP14), AQ17248 for *Apis mellifera* vitellogenin receptor isoform X2, XM_012025824.1 for *Microplitis demolitor* fibrilin 1 like (Md fibrilin 1 like), CP012526.1 for *Microplitis demolitor* cell death abnormal protein, XM_008552822.1 for *Microplitis demolitor* protein kinase C binding protein, AC242691.1 for *Apis mellifera* precursor, EF71640.1 for *Trichoplusia ni* putative tyrosine phosphatase, AE014296.5 for *Nasonia vitripennis* glucose dehydrogenase, FQ312027.1 for *Cotesia congregata* EP1 like protein, AJ632329.1 for *Apis florea* (Af) low density lipoprotein receptor, EF710637.1 for *Drosophila melanogaster* (Dm) ecdysone inducible gene E1, and XM_008565703.1 for *Apis florea* (Af) glycosylphosphatidylinositol.

TSP ORF was interrupted by one intergenic region and 3 introns (Fig 2A). Furthermore, a gene-finding program identified the following two proteins: *Cp-TSP14* (1–3638 in nucleotide sequence) and *Cp-TSP13* (2358–3638) (S1 Fig). *Cp-TSP13* was predicted to possess 4 exons (E2-E3-E4-E5). This was confirmed by its PCR product and subsequent DNA sequence analysis (Fig 2B). Interestingly, a PCR of *Cp-TSP14* against parasitized larvae of *P*. *xylostella* (P1-P8 mixture) produced two PCR products consisting of different exons after DNA sequence analysis: Cp-TSP14a (E1-E4-E5) and Cp-TSP14b (E1-E3-E4-E5). The domain structures of these three similar genes were compared (Fig 2C). Only Cp-TSP13 possessed signal peptide in the amino terminus. However, their C-terminal sequences were identical, showing a typical Cys-motif similar to TSP14 of *M*. *croceipes* (Fig 2D). These Cys-motif genes were clustered and distantly associated with other related genes (Fig 2E).

### *Cp-TSP13* is expressed at all stages of parasitized larvae

**Fig 3:**
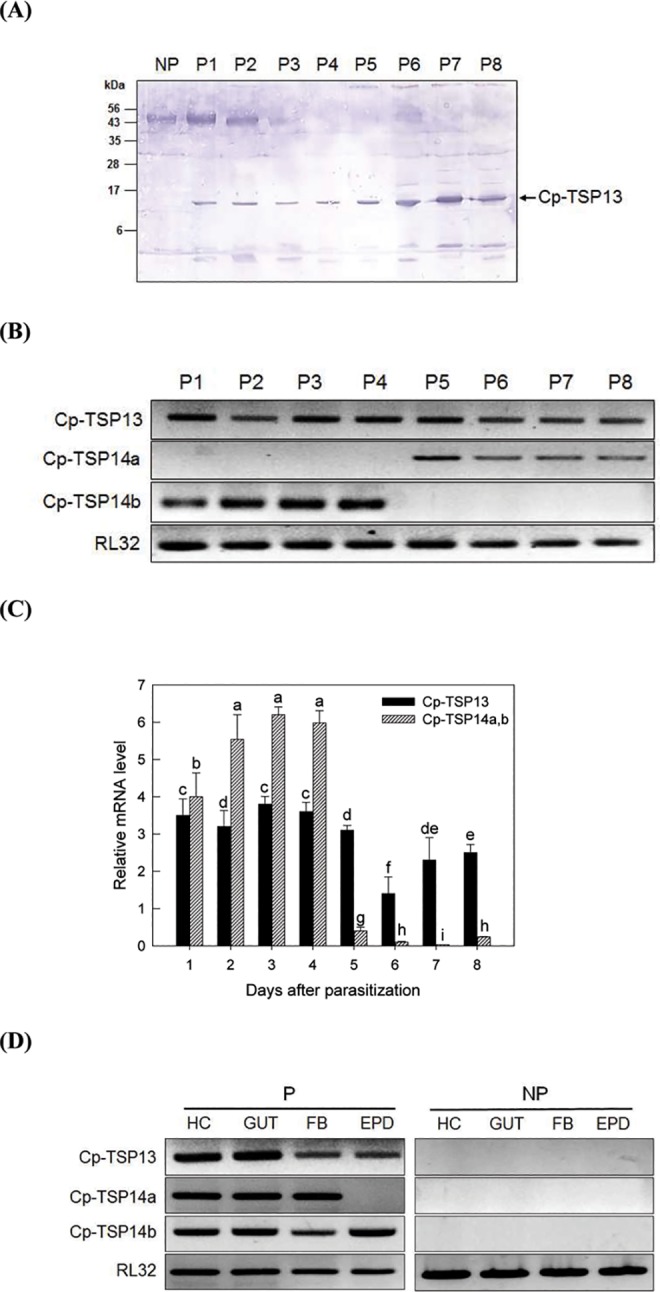
Differential expression of three Cys-motif genes *Cp-TSP13*, *Cp-TSP14a*, and *Cp-TSP14b* in parasitized (P) larvae of *P*. *xylostella*. ‘P1-P8’ indicates days after parasitization. ‘NP’ represents nonparasitized last instar larvae. (A) Western analysis of Cp-TSP13 from total protein extracts of whole body using a polyclonal antibody against rCp-TSP13 raised in rabbit. Proteins were separated on 10% SDS-PAGE. Each lane was equally loaded with 50 μg proteins. (B) RT-PCR analysis of three Cys-motif genes during parasitism. *RL32* is a ribosomal protein gene used as positive control for gene expression in RT-PCR. (C) RT-qPCR analysis of three Cys-motif genes during parasitism. Different letters above standard deviation bars indicate significant difference among means at Type I error = 0.05 (LSD test). (D) RT-PCR analysis of three Cys-motif genes in different tissues of P7 larvae. ‘HC’ for hemocytes, ‘GUT’ for midgut, ‘FB’ for fat body, and ‘EPD’ for epidermis.

Gene-specific primers (Table 2) were designed to monitor the expression levels of the following three Cys-motif genes: *Cp-TSP13*, Cp-TSP14a, and Cp-TSP14b. Parasitized larvae lived for 8 days (P1-P8). Then they pupated at 25°C. *Cp-TSP13* was expressed at all ages of parasitized larvae at protein level analyzed by a western blotting (Fig 3A) and mRNA level (Fig 3B). Some nonspecific bands at western blotting were observed at 45 kDa and small (< 6 kDa) sizes. These nonspecific bands were also found in NP, suggesting a cross-reactivity of the polyclonal antibody. RT-qPCR also supported that *Cp-TSP13* maintained significant levels of expression at all stages of parasitism (Fig 3C). Interestingly, two splicing variants of *Cp-TSP14* were alternatively expressed (Fig 3B). *Cp-TSP14b* was expressed in the first 4 days, while *Cp-TSP14a* was expressed after the 4 days. *Cp-TSP14*s were more highly expressed at young stages than *Cp-TSP13* (Fig 3C). However, their expression levels were decreased at later parasitism stages. *Cp-TSP13* maintained significantly high expression levels until the end of parasitism period. Both variants of *Cp-TSP14* were different in expression pattern in tissues. *Cp-TSP13* and *Cp-TSP14b* were expressed in all tissues of P7 larvae. However, *Cp-TSP14*a was not expressed in the epidermis (Fig 3D).

**Table 2:** Sequences of *Cp-TSP13/14* gene primers used in this study.

Purpose	Primers	Sequences	Amplicon size (bp)	Annealing temp (°C)
RT-PCR	Cp-TSP13 FP	ATGACAATTTGCGTTGTTTAT	342	52
	Cp-TSP RP	CCATTTAGCCCACGGTTTCA		
qRT-PCR	RT-TSP13FP	ATGGACAATTTGCGTTGTTTATACG	210	50
	RT-TSP13RP	ATGGAAATTAAATGTTTTGAT		
Expression vector	Cp-TSP13 pIZT FP	GATATCGGCATGGAAATTAAATGTTTTGAT	354	52
	Cp-TSP13 pIZT RP	GCTCTAGATTCGTCGTGCCATTTAGCCC		
Alternative splicing	Cp-TSP14 FP	TGGTAGAGAGCGTAAGGAGTTC	376(TSP14a) 288(TSP14b)	53
	Cp-TSP RP	CCATTTAGCCCACGGTTTCA		
	Cp-TSP14a FP	ATTCTTGAGTGTAAAGACGAAGC	198	52
	Cp-TSP14a RP	GAATTCGCCCTTCGGGATCCATG		
	Cp-TSP14b FP	TTTAATGCATCGATAGATTCTG	142	52
	Cp-TSP RP	CCATTTAGCCCACGGTTTCA		
Inverse PCR				
Excision site	rCp-TSP13 FP1	AAACTCCATACATTTTAGACTAC	426	50
	rCp-TSP13 RP1	CAAAAGCATTATACAAAGCA		
Non-Excision site	rCp-TSP13FP2	GTACAAATTTCAGTCACATAATT	425	50
	rCP-TSP13RP2	ATTGCTTATTTAGGCTTGACAT		

### *Cp-TSP13* is encoded in the CpBV genome

**Fig 4:**
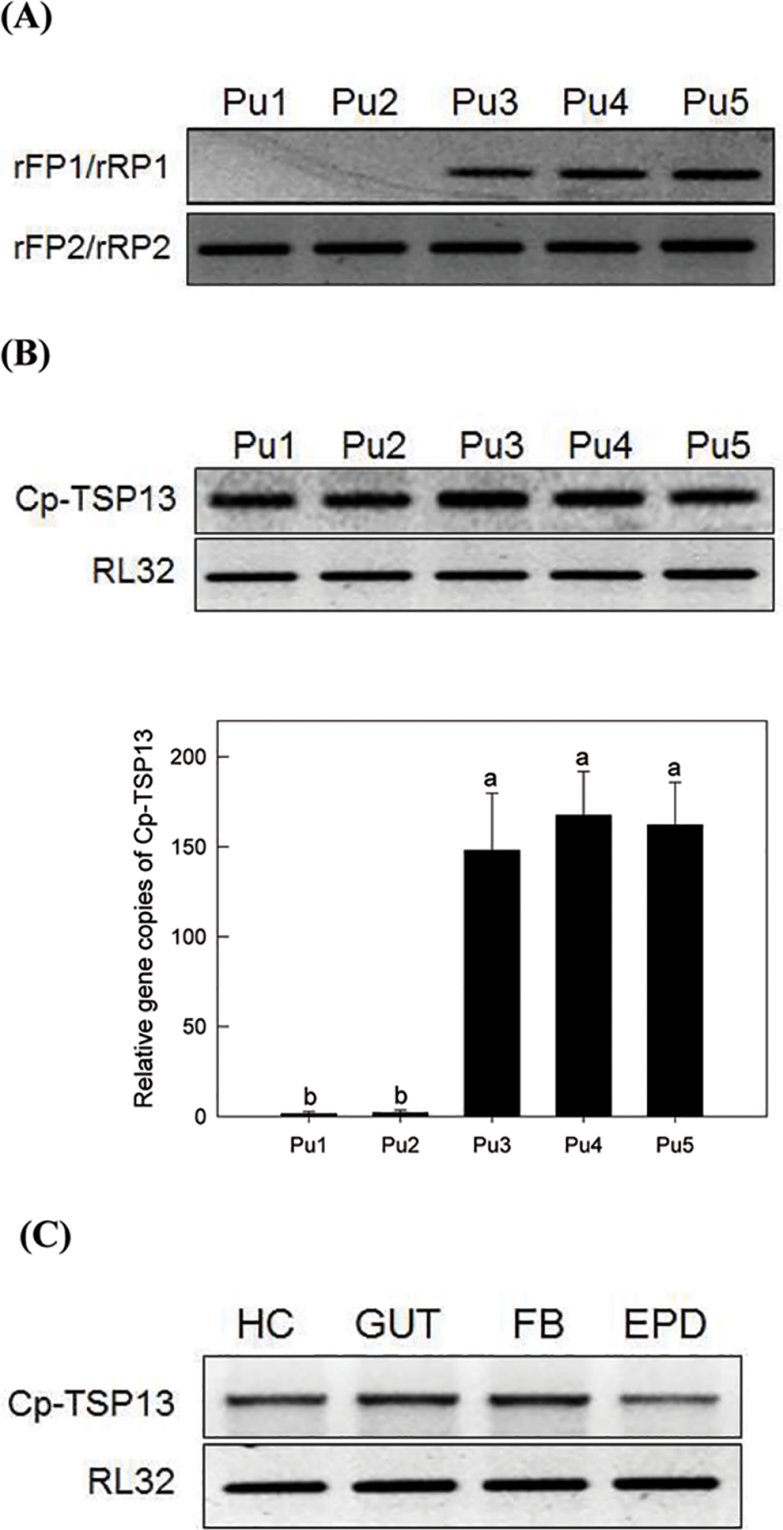
Replication of CpBV-NS1 containing Cp-TSP13. (A) CpBV-NS1 segment excision and rejoining at ER site (see Fig 1C) during pupal period: ‘Pu1-Pu5’ for 1–5 days old pupae. Inverse PCR was performed using primers of rFP1 and rRP1 (rCp-TSP13FP1 and rCp-TSP13RP1, Table 2) to amplify integration/rejoining site of CpBV-NS1. As a reference, primers of rFP2 and rRP2 (rCp-TSP13FP2 and rCp-TSP13RP2, Table 2) were used to amplify non-rejoining site of CpBV-NS1 in PCR. (B) Quantification of Cp-TSP13 in gene content using qPCR. Hexokinase (HEX) was used as a reference gene [79]. Different letters above standard deviation bars indicate significant difference among means at Type I error = 0.05 (LSD test). (C) Expression of *Cp-TSP13* in nonparasitized larvae of *P*. *xylostella* by injection of CpBV virions at a dose of 0.1 female equivalent (FE). Expression was analyzed by RT-PCR. *RL32* is a ribosomal protein gene used as positive control for gene expression in RT-PCR.

Expression of *Cp-TSP13* at early stages of parasitized larvae suggested that *Cp-TSP13* might be encoded in the CpBV genome as in CpBV-NS1 (Fig 1C). An inverse PCR using two gene-specific primers (rCp-TSP13 FP1 and rCp-TSP13 RP1 in Table 2) indicated that the excision and rejoining of the segment occurred at late pupal stages of *C*. *plutellae* (Fig 4A). Quantification of *Cp-TSP13* gene content by qPCR showed its significant amplification at late pupal stages (Fig 4B). When purified CpBV virions were injected into NP larvae of *P*. *xylostella* at 0.1 FE dose, *Cp-TSP13* expression was confirmed in all tested tissues at 12 h after the injection (Fig 4C).

### Recombinant Cp-TSP13 is a secretory protein that enters target cells

**Fig 5:**
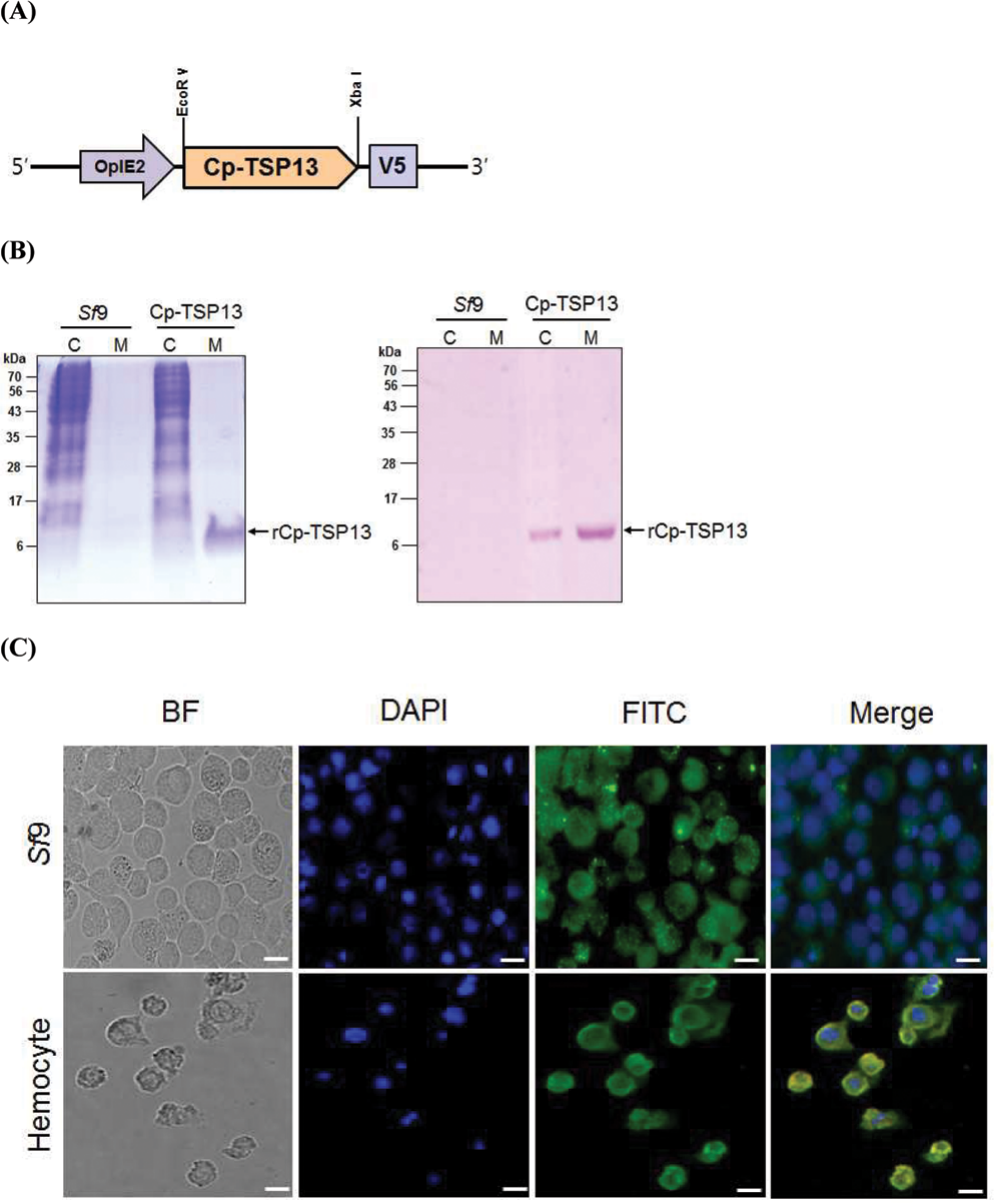
Production of a recombinant Cp-TSP13 (rCp-TSP13) protein in *Sf*9 cells using a eukaryotic expression vector. A recombinant expression vector pIZT/V5-Cp-TSP13 was constructed under a baculovirus immediate early promoter (OpIE2). (A) Overexpressing of rCp-TSP13 in *Sf*9 cells. Proteins were separated into cellular extract (‘C’) and cultural medium (‘M’). ‘*Sf*9’ represents overexpression using empty vector pIZT/V5. ‘Cp-TSP13’ represents overexpression using recombinant vector pIZT/V5-Cp-TSP13. Left panel shows Commassie staining, while right panel shows its western blot result using V5 antibody. (B) Entry of rCp-TSP13 to target cells. rCp-TSP13 was overlaid on monolayer of target cells. Sf9 cells and *P*. *xylostella* hemocytes were used as target cells. Cells were observed under a fluorescence microscope (DM 2500, Leica, Wetzlar, Germany) at bright field (BF) mode for cell contour, DAPI mode for nuclei, or FITC mode for rCpBV-TSP13. No FITC signal was detected in untreated Sf9 cells or hemocytes. Merge mode is the combination of DAPI and FITC pictures. Scale bar: 10 μm.

A recombinant Cp-TSP13 protein was produced in *Sf*9 cells using a eukaryotic expression vector by transient expression. The recombinant Cp-TSP13 protein was detected mostly in the cultured media. It was much less detected in the cell extracts (Fig 5A). Produced recombinant proteins were overlaid onto *Sf*9 cells or hemocytes of *P*. *xylostella* (Fig 5B). Cp-TSP13 entered both cell types. It was located at the cytosol based on immunofluorescence assay, in which DAPI (blue) staining nuclei was clearly distinct from FITC staining Cp-TSP13.

### Translation-inhibitory activity of Cp-TSP13

**Fig 6:**
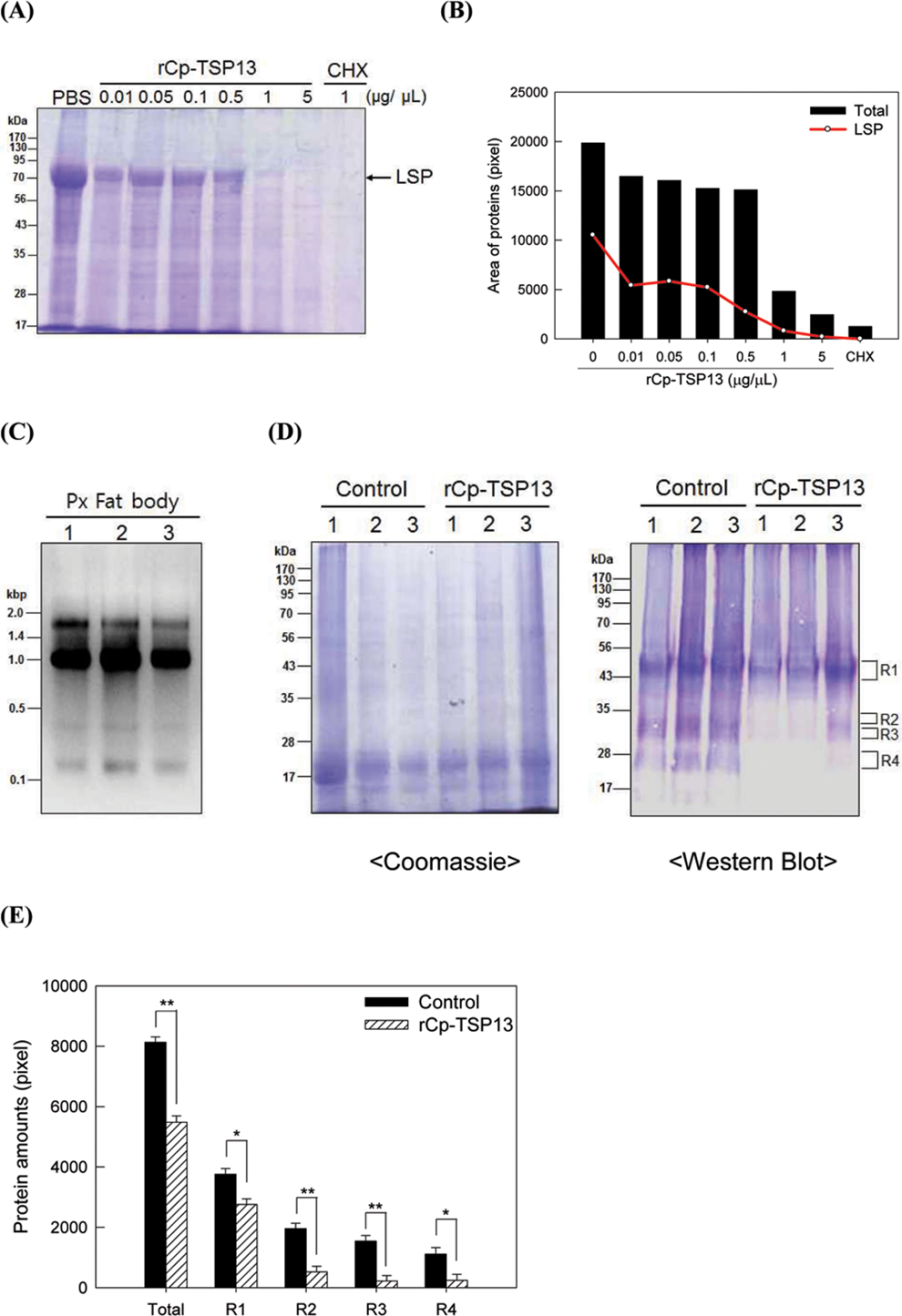
Inhibitory activity of a recombinant Cp-TSP13 (rCp-TSP13) on translation of host mRNAs of *P*. *xylostella*. (A) Inhibition of protein synthesis by rCp-TSP13 in fat body organ culture system. Cycloheximide (CHX) was used as positive control for translation inhibition activity. LSP represents larval storage protein of *P*. *xylostella*. (B) Quantification of newly synthesized proteins in *in vitro* organ culture by densitometry. All three replications were applied for quantification with different independent runs. (C) RNA extraction from fat body of *P*. *xylostella* for *in vitro* translation (IVT) assay using rabbit reticulocyte lysate. Numbers indicate three replications. (D) IVT assay with 1 μg of rCp-TSP13 protein in each reaction (200 μL). Four regions (‘R1-R4’) indicate four newly synthesized protein bands in IVT. (E) Quantification of R1-R4 bands on western blot in IVT assay by densitometry. Asterisks above standard deviation bars indicate significant difference among means at Type I error = 0.05 (*) or 0.01 (**) (LSD test).

Cys-motif genes such as VHv1.4, VHv1.1, and Mc-TSP14 possess HTIF function [40,41]. To test the HTIF activity of Cp-TSP13 due to its similarity to HTIFs, recombinant protein of Cp-TSP13 was incubated with fat body of NP larvae of *P*. *xylostella*. In the control, fat body synthesized and secreted the proteins into the culture media (first lane named PBS, Fig 6A), in which larval storage protein (LSP) was predominantly synthesized and secreted [44, 46]. However, the addition of recombinant Cp-TSP13 protein reduced the amount of proteins secreted into the culture media from the fat body. The inhibitory activity of recombinant Cp-TSP13 against LSP was dose-dependent especially at 0.1–5 μg/μL and as significant as that of cycloheximide (a eukaryotic translation inhibitor) (Fig 6B).

To further analyze the inhibitory activity of Cp-TSP13, rabbit reticulocyte lysate *in vitro* translation (IVT) assay was conducted. Three replicates of total RNA samples were prepared from the fat body of NP larvae (Fig 6C). In the control, the IVT system produced several proteins grouped into R1-R4 (Fig 6D). However, the addition of recombinant Cp-TSP13 into RNA extracts significantly reduced the translation efficiencies of all protein groups (Fig 6E).

### Suppression of cellular immunity by Cp-TSP13

**Fig 7:**
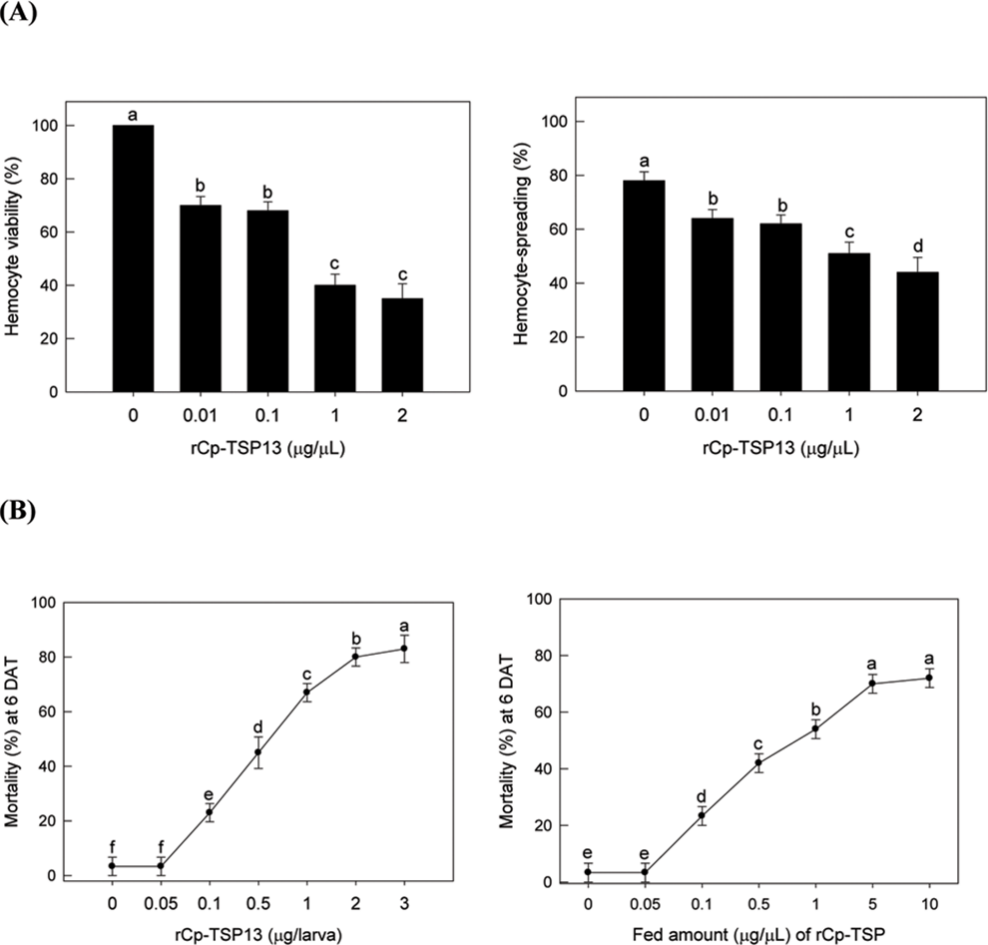
Cytotoxicity and insecticidal activity of recombinant Cp-TSP13 (rCp-TSP13) against *P*. *xylostella* larvae. (A) Left panel: cytotoxicity assay of rCp-TSP13 against hemocytes of *P*. *xylostella* by incubation for 24 h at 25°C. Cell survival was assessed through dye-exclusion assay [76]. Right panel: inhibition of hemocyte-spreading behavior [30] by incubating with different concentrations of rCp-TSP13 for 30 min at 25°C. (B) Insecticidal activity. Left panel: hemocoelic injection of different concentrations of rCp-TSP13 to third instar larvae. Right panel: feeding treatment by leaf-dipping into different concentrations of rCp-TSP13 and feeding to third instar larvae. Each treatment was replicated three times with 10 larvae per replication. Mortality was measured at 6 days after treatment (DAT). Different letters above standard deviation bars indicate significant difference among means at Type I error = 0.05 (LSD test).

The effect of Cp-TSP13 on hemocytes was assessed by their spreading-behavior and cell viability (Fig 7). A short term exposure (< 40 min) of hemocytes to recombinant Cp-TSP13 significantly (*p* < 0.05) inhibited the hemocyte-spreading behavior in a dose-dependent manner (Fig 7A). A longer exposure (~ 12 h) of hemocytes to recombinant Cp-TSP significant (*p* < 0.05) impaired the cell viability based on dye-exclusion assay.

### Developmental retardation of *P*. *xylostella* larvae by Cp-TSP13

Injection or feeding of recombinant Cp-TSP13 to NP larvae of *P*. *xylostella* significantly (*p* < 0.05) prevented the development to pupal stage (Fig 7B). Increased amounts of recombinant Cp-TSP13 resulted in significant mortalities in both injection and feeding assays. For injection analysis, the median lethal dose (LD_50_) of Cp-TSP13 was found to be 582.13 ng/larva (95% CI: 312.54~782.67). For feeding assay, the median lethal concentration (LC_50_) of Cp-TSP13 was found to be 1.23 μg/μL (95% CI: 0.68~2.11).

## Discussion

Cys-motif genes have been identified from IVs. They play critical roles in host regulation by suppressing immune responses and inhibiting host protein synthesis by acting as HTIFs [39,40,48]. A similar Cys-motif gene has been identified in teratocytes of a braconid wasp *M*. *croceipes*. It mediates similar host regulation [49]. BVs are known to have Cys-rich proteins. However, their molecular structures are not similar to those of Cys-motif proteins that act as HTIFs. This study identified three Cys-motif genes encoded in a BV, CpBV. They were found to be one (Cp-TSP13) of Cys-motif genes acting as HTIF.

*Cp-TSP13* gene was located in *Cp-TSP14*. *Cp-TSP13* and *Cp-TSP14* transcripts shared C-terminal two exons (E4-E5). RT-PCR products of *Cp-TSP14* did not contain *Cp-TSP13*, suggesting that alternative choice of transcriptional start may result in these two different genes. *Cp-TSP14* mRNA precursor might be further processed by alternative splicing that two variants (*Cp-TSP14a* and *Cp-TSP14b*) were produced. The two variants were identified by comparing cDNA and genomic DNA sequences. These two alternative splicing variants were mutually exclusive in expression patterns during parasitism. *Cp-TSP14b* was expressed during early parasitism, but *Cp-TSP14a* was expressed during late stage. In contrast, *Cp-TSP13* was constitutively expressed during all parasitic stages. Moreover, Cp-TSP13 protein levels were increased with the development of parasitized larvae. Thus, at least three different transcripts could be derived from *Cp-TSP14* gene construct. All three different proteins shared C-terminus containing conserved six cysteine residues, suggesting a physiological significance of the conserved Cys motif in parasitism (see below). Alternative splicing is usually used to expand limited genetic resources, especially under positive selection pressure in eukaryotes, including insects [50]. Down syndrome cell adhesion molecule (Dscam) of arthropod has been well known to be able to produce multiple isoforms ranging from 4,212 to 38,016 by alternative splicing [51]. Variable isoforms of Dscam contributes to axon guidance in neuronal wiring in *Drosophila* [52]. They function as immunological receptors against various pathogens [53]. Serine proteinase inhibitor (serpin) is another example of alternative splicing that produces hyper-variable isoforms to meet various serine proteinase targets [54]. Thus, alternative splicing variants (*Cp-TSP14a* and *Cp-TSP14b*) and alternative choice of transcriptional starts (*Cp-TSP14*s and *Cp-TSP13*) might be used as strategies for parasitic factor to expand their targets to regulate host physiological processes.

Three *Cp-TSP* genes are clustered into Cys-motif family. This gene family was originally established for 10 genes encoded by *Campoletis sonorensis* ichnovirus [55]. A similar Cys-motif protein TSP14 in another parasitic factor was also detected from teratocytes of *M*. *croceipes* [56]. This study provided additional Cys-motif genes derived from bracovirus. Therefore, Cys-motif genes are shared by both BV and IV as well as teratocytes. Since BV and IV have independent origins [14], Cys-motif genes could be regarded as product of another convergent evolution. Cp-TSP13 is also homologous to TSP14 of *M*. *croceipes* expressed in teratocytes. This current study also revealed that Cp-TSP13 was expressed in venom gland and female adults, but not in male adults, indicating that *Cp-TSP13* encoded in CpBV proviral genome could be expressed in wasp and lepidopteran hosts as venom protein and viral protein, respectively. Alternatively, its duplicated gene encoded in the wasp genome other than CpBV proviral locus may be expressed in venom gland. On the other hand, a comparative study for three parasitic factors of venom, teratocytes, and PDV has suggested that there is no overlap among their expressed genes in *M*. *demolitor* [9]. However, BV genes functioning in pathogenicity are derived from nudivirus-like ancestor or from host or other pathogens through lateral gene transfer. Subsequent duplication and diversification of these genes might have occurred under selective selection pressure to increase successive parasitism [8]. *Cp-TSP13* and related Cys-motif genes are clustered into a unique group by our current phylogenetic analysis, suggesting its ancestral nudiviral origin. Indeed, CpBV-NS1 encoding Cp-TSP13 is surrounded with several transposable elements and nudiviral genes (S1 Table).

Genome sequencing of CpBV-carrying wasp *C*. *plutellae* has allowed the identification of *Cp-TSP13* and *Cp-TSP14* variants in CpBV genome. A novel viral segment at size of 33.4 kb surrounded by PDV-characteristic wasp integration motif (WIM) has been recognized by nudivirus-like tyrosine recombinase to produce circularized segments [20,57,58]. This segment was not identified from previous genomic analyses using plasmid-capture system [59] or Illumina HiSeq [34]. Similar approaches to sequence whole genomes identified new additional PDV segments [17,23]. The new CpBV segment was validated by qPCR, demonstrating its replication and resulting in the amplification of Cp-TSP13 gene content. In addition, an inverse PCR at the excision and rejoining site confirmed its excision from the wasp genome and insertion into viral particles.

Both *Cp-TSP14* variants lack E2 exon that contains signal peptides, suggesting that these two variants may play their physiological roles within target cells. In contrast, Cp-TSP13 starting with E2 is secreted into hemolymph, suggesting that it could be transported to other cells within the host. Our overlay assay using a recombinant Cp-TSP13 protein indicated that Cp-TSP13 could enter cells and reside at the cytosol. It was revealed that Cp-TSP13 could inhibit the translation of host mRNAs. *In vitro* cultured fat body was impaired in secreting newly formed proteins to culture media when it was incubated with Cp-TSP13. The inhibitory activity of Cp-TSP13 mimicked the effect of a well-known eukaryotic translation inhibitor cycloheximide. In addition, IVT assay using rabbit reticulocyte lysate confirmed that Cp-TSP13 could directly inhibit host mRNAs. Translation-inhibitory activity of Cys-motif proteins has been discovered in VHv1.4 and VHv1.1 of CsIV against mRNAs of *H*. *virescens* [40]. These two Cys-motif protein can enter target cell and selectively inhibit mRNAs, in which arylphorin (a larval storage protein) is likely to be the main target [39]. Parasitized or CsIV-infected *H*. *virescens* larvae were lack of arylphorin in the plasma. However, the level of arylphorin was not changed in the control or the treatment. Shelby and Webb [48] have discovered selective inhibition of viral HTIF by inhibiting translation of storage proteins and juvenile hormone (JH) esterase without affecting the translation of transferrin, ferritin, or JH-binding protein. This selective inhibition can be understood in terms of koinobiont life style of the endoparasitoid wasp to supply limited host nutrient resources to growing wasp immatures while host larvae keep their feeding behavior. Later, Kim [40] identified HTIF factors by fractionation of plasma extracted from parasitized larvae and subsequent HTIF bioassay. Two Cys-motif proteins (VHv1.4 and VHv1.1) were found to be able to inhibit the translation of mRNAs extracted from fat body in a dose-dependent manner [40]. Another HTIF was discovered in teratocyte of *M*. *croceipes* with similar translation inhibitory activity especially against arylphorin mRNA [41]. However, the molecular action on how these HTIFs inhibited translation was unknown. Another type of HTIFs was identified in CpBV. Two homologous proteins of CpBV15α and CpBV15β were found to be able to specifically inhibit host gene expression at post-transcriptional level [43]. These CpBV HTIFs share sequence homologies with eukaryotic translation initiation factors (eIFs). CpBV15β is homologous to eIF4G at the eIF4A-binding site. An immunoprecipitation assay has revealed that CpBV15β can sequester eIF4A, a protein that plays a crucial role in scanning of cap-binding protein during formation of translation initiation complex [46]. eIF4A can unwind the secondary structure of 5’-untranslational region (5’ UTR) of mRNA [60,61]. Different mRNAs have various degree of secondary structure complexity at the 5’UTR. Translation of any mRNAs that are heavily dependent on the activity of eIF4A to unwind their secondary structure at the 5’UTR can be inhibited efficiently by CpBV15β. This has been demonstrated by switching 5’UTRs between target and non-target mRNAs [62] and by point mutation to generate different degrees of 5’UTR complexity [63]. CpBV15α is homologous to eIF5. It interacts with eIF2 to inhibit the formation of 80S ribosome initiation complex for translation [45]. Without information on the molecular action of Cys-motif proteins including Cp-TSP13, we analyzed the domain structures of Cp-TSP13 and found that it had granulin-like domain as well as EGF motif. Granulins are a family of secreted and glycosylated peptides that are cleaved from a single precursor protein with 7.5 repeats of a highly conserved 12-cysteine granulin/epithelin motif [64]. Large progranulin is post-translationally processed into different granulins to mediate various physiological processes such as cell cycling, wound healing, and brain development [65]. In insects, granulin-like molecules have been reported in locust (*Schistocerca gregaria*), mosquito (*Aedes albopictus*), and moth (*Manduca sexta*) with cell proliferating activity [66]. With respect to gene expression control, granulin is known to be able to activate PKC pathway and Toll-like receptor 9 signaling or interact with transcriptional factor such as STAT3 in breast cancer cells [67,68]. These molecular interacting properties of granulin suggest that Cp-TSP13 may interact with translational factors. Disintegrin domain was found in Cp-TSP14a. Snake venom disintegrins work by countering blood clotting steps, inhibiting the clumping of platelets by antagonizing integrins that can mediate cell-cell and cell-extracellular matrix interactions serving as the final common pathway, leading to aggregation via forming platelet-platelet bridges [69]. In *Bombyx mori*, a disintegrin domain is linked to metalloprotease. It plays a role in digesting and remodeling tissues during metamorphosis [70]. Interestingly, this current study showed that Cp-TSP13 sharing C terminal domain of Cp-TSP14 also possessed immunosuppressive activity.

This study provides a new Cys-motif gene in PDV. *Cp-TSP13* is encoded in a new CpBV segment and expressed in parasitized *P*. *xylostella*. It is also expressed in venom gland of *C*. *plutellae*. In *M*. *croceipes*, this Cys-motif is expressed in teratocytes [41]. These suggest a horizontal gene transfer of Cys-motif gene among three parasitic factors of venom, teratocyte, and PDV in BV-associated endoparasitoid wasps. This horizontal gene transfer has been regarded as an evolutionary mechanism for PDV to acquire virulence factors [23].

In summary, a recombinant protein of Cp-TSP13 exhibited potent insecticidal activity through oral administration. Its HTIF activity may partially explain host regulatory activity of Cp-TSP13 as other Cys-motif proteins [71,72]. Furthermore, i*n vitro* assays showed its insecticidal activity by exhibiting both immunosuppressive and cytotoxic effects. The insecticidal activity of Cp-TSP13 suggests that CP-TSP13 could be used a novel gene target to develop transgenic crops in the agricultural industry.

## Materials and Methods

### Insects and parasitization

*P*. *xylostella* larvae were reared at 25 ± 1°C and 16:8 h (L:D) photoperiod with cabbage leaves. Adults were fed with 10% sucrose. Late second instar larvae were parasitized by *C*. *plutellae* at 1:2 (wasp: host) density for 24 h and reared under the same environmental conditions. After emergence, adult wasps were allowed to mate for 24 h. After that, they were used for parasitization.

### Interrogation of DBM genome database and *in silico* analysis of Cp-TSP13

A transcriptome of a venom gland of *C*. *plutellae* was obtained from Dr. Daeweon Lee (Kyungsung University, Busan, Korea). One annotated transcript was TSP14 of *M*. *croceipes*. With its partial sequence, full genome of *C*. *plutellae* was screened using a genome browser (http://220.69.246.37/gbrowse2/). A highly matched sequence was located in scaffold 67 with size of 715,386 bp. Open reading frames (ORFs) of scaffold 67 were predicted using a gene-finding program (FGENESH, http://linux1.softberry.com/berry.phtml?topic=fdp.htm). Excision sites appeared to be conserved among different segments and even different PDV species [47]. The conserved sequence (wasp integration motif (WIM: AGCTTT+boundary sequence)) was searched in scaffold 67 using SeqEdit program of DNAstar (Version 5.02, DNAstar Inc., Madison, WI, USA). Putative WIM sites were aligned with previous known WIM sequences using HMMER program (http://hmmer.janelia.org/). The resulting new segment was named CpBV-NS1. To validate the excision motif of CpBV-NS1, inverse PCR (iPCR) primers (rCp-TSP13 FP1 and rCp-TSP13 RP1, Table 2) were designed around the putative WIM. Using gDNAs isolated from different developmental pupal stages of *C*. *plutellae*, iPCRs were performed using 35 cycles of an array of reactions of the following: 94°C for 30s, 52°C for 30s, and 72°C for 1 min.

### RNA extraction and cDNA preparation

Total RNAs of parasitized (P) and nonparasitized (NP) larvae of *P*. *xylostella* were extracted using Trizol reagent (Invitrogen, Carlsbad, CA, USA) according to manufacturer’s instructions. For RNA extraction, 20 young larvae (1st-3rd instar), three 4th instar larvae, and one 5th instar larva were used. Extracted RNAs were treated with RNase-free DNase (Bioneer, Seoul, Korea) to degrade any genomic DNA contamination. RNA extract (1 μg per reaction) was incubated at 70°C for 3 min and used to synthesize cDNA using RT-premix (Intron Biotechnology, Seoul, Korea) containing oligo dT primer and reverse transcriptase.

### RT-PCR and quantitative RCR (qPCR)

Synthesized cDNAs were used for PCR amplification with *Cp-TSP13* forward and reverse primers (Cp-TSP13 FP and Cp-TSP RP, Table 2). After an initial denaturation at 94°C for 5 min, RT-PCR was performed with 35 cycles of denaturation at 94°C for 30 s, annealing at 50°C for 30 s, and extension at 72°C for 1 min. The PCR reaction was ended with an extension step at 72°C for 10 min. SYBR Green Realtime PCR master mixture (Toyobo, Osaka, Japan) was used for qPCR with a 7500 real time PCR system (Applied Biosystems, Foster City, CA, USA) according to the manufacturer’s instruction. The reaction mixture (20 μL) included 5 pmol of forward and reverse primers as described in RT-PCR using 50 ng of template cDNA. After activating Hot-start Taq DNA polymerase at 94°C for 15 min, the reaction was performed with 35 cycles of 30 sec at 94°C, 30 sec at 50°C, and 1 min at 72°C with a final extension for 5 min at 72°C. Fluorescence values were measured and amplification plots were generated in real time with an Exicycler^TM^ program. In each run, single PCR products were confirmed by their melting curves. Quantitative analysis of amplification was performed using comparative C_T_ method [73]. For an endogenous control ribosomal protein (RL32) primers (FP: 5′-ATGCCCAACATTGGTTACGG-3′, RP: 5′-TTCGTTCTCCTGGCTGCGGA-3′) was used.

### Purification of CpBV virions

CpBV virions were collected from ovaries of *C*. *plutellae* as described by Bae and Kim [29]. Briefly, one-day old females were collected and dissected to obtain ovaries with phosphate-buffered saline (PBS, pH 7.4). Ovarian calyx was teased to release CpBV viral particles to surrounding PBS. The PBS solution containing the virions was filtered through a membrane (pore size: 0.50 μm, Pall Life Sciences, Seoul, Korea). The filtrate was centrifuged at 13,500 x *g* for 20 min at 4°C. The pellet was resuspended with 1 μL of fresh PBS per ovary extract. The viral quantity was expressed as female equivalent (FE). One FE of CpBV represented a whole viral extract obtained from a pair of ovaries.

### Microinjection and expression analysis

CpBV was injected at a dose of 0.1 FE into larval hemocoel of NP *P*. *xylostella* through dorsal intersegmental membrane using Ultra Micropump with SYS-microcontroller (World Precision Instruments, Sarasota, FL, USA). Microinjection was performed under a stereomicroscope (Olympus S730, Tokyo, Japan). After injection, larvae were fed cabbage and cultured at 25°C. Gene expression was analyzed by RT-PCR using gene-specific primers described above.

### Construction of recombinant expression vector expressing Cp-TSP13

*Cp-TSP13* ORF was cloned by PCR with a high fidelity Taq polymerase (Platinum^®^ Taq DNA Polymerase, Invitrogen) and gene-specific primers containing *Not*I and *Sac*II restriction sites (Cp-TSP13 pIZT FP and Cp-TSP13 pIZT RP, Table 2). Thermal conditions for PCR were 95°C for 3 min for pretreatment, followed by 35 cycles of 95°C for 1 min, 55°C for 45 sec, 72°C for 2.4 min, and extension at 72°C for 7 min. PCR products were digested with *Not*I and *Sac*II restriction enzymes and cloned into pIZT/V5-His vector (Invitrogen).

### Cell transfection and recombinant protein preparation

Recombinant pIZT/V5-CpTSP13 was used for protein production in *Sf*9 (IPLB-Sf21-AE) cell line derived from *Spodoptera frugiperda* pupal ovarian tissues by cationic lipid-mediated transfection method using X-treme GENE 9 DNA transfection reagent (Roche, Mannheim, Germany). Briefly, 2 x 10^6^ *Sf*9 cells in serum-free TC100 insect culture medium (Hyclone, Daegu, Korea) were seeded onto T-25 cm^2^ tissue culture flasks (Nunc, Roskilde, Denmark) at 4 h prior to transfection. Recombinant DNA (2.0 μg) was mixed with 5 μL of transfection reagent following the manufacturer’s instruction for standard transient transfection. Empty vector pIZT/V5 was used for control transfection. After 48 h of incubation at 28°C, cells were washed with fresh PBS and resuspended in PBS. After ultrasonication at 4°C for 30 sec, cell extract was centrifuged (1,200 x *g*, 10 min, 4°C) and the supernatant was used as cellular protein extract. Subsequent SDS-PAGE and western analyses were performed using method described by Kumar and Kim [74]. To collect proteins in culture medium, culture media were separated by pipetting from cells and then subject to centrifugation at 800 x *g* for 3 min at 4°C to obtain the supernatant. The resulting recombinant Cp-TSP13 (rCp-TSP13) was quantified by Bradford [75] method using BSA standard. Purified protein was used to raise a polyclonal antibody in a rabbit.

### Cp-TSP13 cell entry and immunofluorescence assay

Ten μL of rCpTSP-13 (5 μg/μL) in PBS was overlaid on *Sf*9 cells (2 x 10^6^ cells/sample) for 1 h at 25°C. Treated cells were washed twice in PBS by centrifugation at 800 x *g* for 5 min. Cells were then fixed with 4% paraformaldehyde for 5 min at 25°C. After washing once with PBS, cells were permeabilized with 0.2% Triton X-100 in PBS for 5 min at 25°C followed by treatment with 90% ice cold acetone for 2 min at -20°C. Cells were washed once in PBS and blocked with 1% BSA in PBS for 60 min at 25°C. After washing once in PBS, cells were incubated with primary antibody (mouse monoclonal anti-V5 antibody, Invitrogen; 1:1,000) in PBS at 25°C for 1 h. After washing three times, secondary antibody (anti-mouse IgG conjugated with fluorescein isothiocyanate (FITC) (Sigma-Aldrich Korea; 1:5,000)) in PBS was added and incubated at 25°C for 1 h. After washing twice, cells were incubated with 4'-6-diamidino-2-phenylindole (DAPI, diluted 1,000 times in PBS of 1 mg/mL stock, Thermo Scientific, Rockford, IL, USA) for nucleus staining. After washing twice in PBS, cells were incubated with 0.04% trypan blue for 10 min to quench proteins on the cell surface and then observed under a fluorescence microscope (DM2500, Leica, Wetzlar, Germany) at 400 x magnification to determine protein localization.

### *In vitro* organ culture and HTIF assay

Fat body from NP larvae of *P*. *xylostella* was collected in 0.5 mL of PBS. The fat body was pre-incubated in PBS for 30 min to remove intracellular preformed secretory proteins and to deplete endogenous amino acid pool. The resulting fat body was overlaid with 100 μL of TC-100 insect medium containing different concentrations of rCp-TSP13 or cycloheximide (1 μg/μL) and allowed to newly synthesize and release proteins into culture medium at 25°C for 24 h. Proteins released into the culture medium were separated on 10% SDS-PAGE and stained with Coomassie brilliant blue.

### *In vitro* translation assay using rabbit reticulocyte lysate

To test inhibitory effect of Cp-TSP13, rabbit reticulocyte lysate (Flexi^®^ Rabbit Reticulocyte Lysate System, Promega Korea, Seoul, Korea) *in vitro* translation assay system was used. rCp-TSP13 was treated with 1 μL of RNase inhibitor (4 units/μL, Promega Korea) and 5 μL of *P*. *xylostella* fat body RNA (1 μg/μL, denatured at 65°C for 3 min). The mixture was pre-treated at 30°C for 10 min. Enzyme mixture consisted of 30 μL of rabbit reticulocyte lysate, 0.5 μL of 1 mM complete amino acid mixture (minus lysine), 2 μL of 25 mM magnesium acetate, 1.4 μL of 2.5 mM KCl, and 0.5 μL of 100 mM dithiothreitol. After the addition of both mixtures, translation reaction was carried out at 30°C for 120 min by adding 1 μL of biotinylated lysine-containing Transcend™ tRNA.

*In vitro* translation was analyzed by colorimetric detection. Briefly, biotin-containing translation products were mixed with SDS-denaturing buffer. After boiling for 5 min at 95°C, samples (20 μL per lane) were run on 10% SDS-PAGE. For western blotting, the gels were electro-transferred onto immunoblot^TM^ polyvinylidene difluoride (PVDF) membranes (Bio-Rad, Hercules, CA, USA). After electro-transfer, the membranes were blocked with 5% skim milk at 25°C for 1 h. They were washed three times with 100 mM Tris-buffered saline containing 0.05% Triton X-100 (TBST) and incubated in fresh 15 mL of 5% skim milk containing 6 μL of streptavidin-conjugated alkaline phosphatase (1:3,000 dilutions, Sigma-Aldrich Korea) at 25°C for 60 min. Finally, membranes were washed three times in TBST and incubated with nitroblue tetrazoleum chloride/5-bromo-4-chloro-3-indolyl phosphate (Sigma-Aldrich Korea).

### Hemocyte-spreading assay

Hemocyte-spreading assay was performed in 96-well culture plate (SPL, Pochen, Korea). A 50 μL of reaction mixture in each well consisted of rCp-TSP13 (5 μL) and hemocyte suspension in TC-100 medium (45 μL). Control consisted of the same incubation composition by replacing recombinant protein with TC-100 medium. After mixing the components well, the plates were kept under darkness at 25°C for 40 min. Hemocytes that exhibited pseudopodial extension were counted under a phase contrast microscope (400x, IX70, Olympus). Each measurement used 100 hemocytes. Each treatment had three replicates.

### Cytotoxicity test using dye-exclusion assay

rCp-TSP13 was injected to NP larvae at different doses (0, 0.01, 0.1, 1, 10 μg) per individual. Fourth instar larvae were used in this assay. After the injection, treated larvae were kept at 25°C for 12 h. Hemocytes were then collected into 1.5 mL cold tube, mixed with 0.04% trypan blue dye (1:1, v/v), and incubated at 25°C for 5 min. Hemocytes without absorbing the dye were regarded as living cells (‘dye exclusion method’) [76]. The number was counted with a hemocytometer (Superior, Germany) under a phase contrast microscope (BX41, Olympus). Each dose treatment was independently replicated in three individuals.

### Toxicity assays of rCpTSP13

To assess the insecticidal activity of rCp-TSP13, NP larvae were used for injection and feeding assays. For injection bioassay, different doses (0, 0.01, 0.1, 1, 10 μg/larva) of rCp-TSP13 proteins were injected into hemocoel of the 3^rd^ instar NP larvae. For feeding assay, cabbage was cut into 20 mm in diameter and soaked in recombinant protein solutions containing different concentrations (0, 0.01, 0.1, 1, and 10 μg/mL). The 3^rd^ instar larvae were fed treated cabbages for 24 h. After injection or feeding, all test larvae were fed fresh cabbage until death or pupation. Each treatment used 10 larvae (three replicates per treatment). Mortalities in both assays were recorded at 6 days after treatment.

### Statistical analysis

All bioassays were performed in three independent biological replicates. Data (mean ± standard deviation) were plotted using Sigma plot. Means were compared by least squared difference (LSD) test of ANOVA using PROC GLM of SAS program [77] at Type I error of 0.05.

## Supporting Information

S1 Fig Genomic sequence of Cp-TSP genes encoded in scaffold 67 of *C*. *plutellae* genome. Start codons are boxed in black color. Stop codon is boxed in red color. Underlines indicate exons.
(DOCX)

S1 Table Predicted genes encoded in scaffold 67 of *C*. *plutellae* genome. Ten ORFs (ORF30-ORF39) are encoded in CpBV-NS1.
(DOCX)
