# Supplementary material for: Translational Control of Host Gene Expression by a Cys-Motif Protein Encoded in a Bracovirus
Source: PLoS One. 2016 Sep 6;11(9):e0161661. doi: 10.1371/journal.pone.0161661 (PMC5012692; doi:10.1371/journal.pone.0161661)
Supplement: S1 Fig — Start codons are boxed in black color. Stop codon is boxed in red color. Underlines indicate exons. (DOCX) [file pone.0161661.s001.docx]

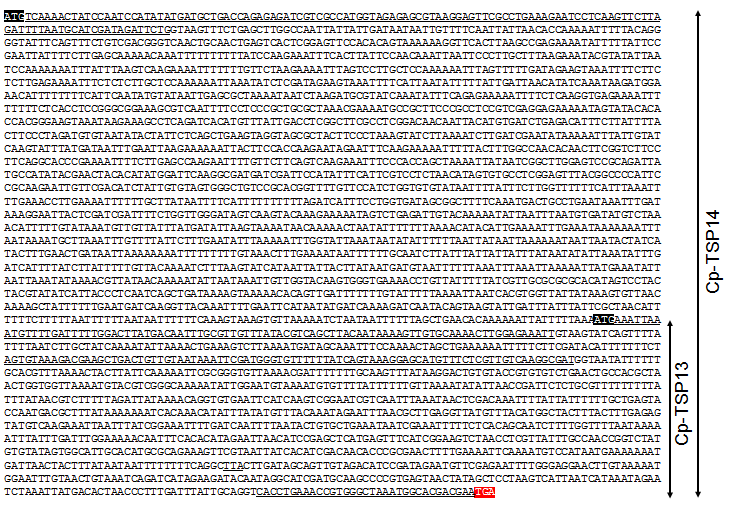


**Fig. S1**. Genomic sequence of Cp-TSP genes encoded in scaffold 67 of *C. plutellae* genome. Start codons are boxed in black color. Stop codon is boxed in red color. Underlines indicate exons.
