# Supplementary material for: Translational Control of Host Gene Expression by a Cys-Motif Protein Encoded in a Bracovirus
Source: PLoS One. 2016 Sep 6;11(9):e0161661. doi: 10.1371/journal.pone.0161661 (PMC5012692; doi:10.1371/journal.pone.0161661)
Supplement: S1 Table — Ten ORFs (ORF30-ORF39) are encoded in CpBV-NS1. (DOCX) [file pone.0161661.s002.docx]

**Table S1**. Predicted genes encoded in scaffold 67 of *C. plutellae* genome. Ten ORFs (ORF30-ORF39) are encoded in CpBV-NS1.

| ORFs | Location | AA (residues) | Predicted gene  (species) | GenBank  accession number | E-value |
| --- | --- | --- | --- | --- | --- |
| ORF1 | 60392←11803 | 169 | Transposon (*Cotesia congregata*) | EU822800.1 | 5.00E-43 |
| ORF2 | 10843→11427 | 100 | LEF-8 (*Cotesia sesamiae)* | HF58473 | 4.00E-04 |
| ORF3 | 16414→16917 | 166 | Xylulose kinase (*Bombyx mori*) | XM_004925200.2 | 7.00E-22 |
| ORF4 | 16901→17797 | 298 | DNA helicase (*Cotesia congregata*) | XN_00820419 | 3.00E-82 |
| ORF5 | 18567→20766 | 56 | ODV orf-E56-1 (*Cotesia congregata*) | FM212913.1 | 2.00E-09 |
| ORF6 | 37175→79893 | 504 | GP1-anchored adhesin-like protein  (*Microplitis demolitor*) | XM_008547805.1 | 0.00E+00 |
| ORF7 | 38194→38595 | 133 | Huntingtin  ( *Microplitis demolitor*) | XM_008547832.1 | 1.00E-74 |
| ORF8 | 38826→39320 | 164 | Huntingtin (*Microplitis demolitor)* | XM_008547832.1 | 1.00E-70 |
| ORF9 | 39510→46028 | 2172 | Huntingtin (*Microplitis demolitor*) | XM-008547832.1 | 1.00E-50 |
| ORF10 | 61274←62185 | 303 | TRIM transposon  (*Drosophila miranda*) | X59239.1 | 3.00E-44 |
| ORF11 | 63092←74038 | 375 | Polydnavirus-associated P-element transposable gene (*Glyptapanteles indiensis*) | EU822800.1 | 0.00E+00 |
| ORF12 | 122862→123329 | 112 | LEF-8 (*Cotesia congragata*) | FM_212912.1 | 5.00E-33 |
| ORF13 | 123570←124223 | 127 | Tigger transposable element (*Acyrthosiphon pisum*) | XM_008180604.1 | 4.00E-104 |
| ORF14 | 128714←129944 | 132 | Small nuclear ribonucleoprotein  (*Microplitis demolitor*) | XM_008552418.2 | 8.00E-51 |
| ORF15 | 132199→137412 | 125 | Elongation of very long chain fatty acids protein 7-like  (*Microplitis demolitor*) | XM_008562371.1 | 4.00E-43 |
| ORF16 | 135241→135687 | 157 | Reverse transcriptase (*Pediculus humanus*) | XM_002431950.1 | 1.00E-11 |
| ORF17 | 147320→148823 | 162 | DNA polymerase (*Microplitis demolitor)* | XM_008555439.1 | 1.00E-05 |
| ORF18 | 156718←165213 | 285 | Elongation of very long chain fatty acids protein 7-like  (*Drosophila melanogaster*) | xm_00856237.1 | 5.00E-153 |
| ORF19 | 164851←165240 | 129 | Small nuclear ribonucleoprotein A (*Microplitis demolitor*) | XM_008555966.1 | 1.00E-85 |
| ORF20 | 207448←207657 | 169 | Kinetochore protein (*Gossypium raimondii*) | XM_012607761.1 | 2.00E-12 |
| ORF21 | 209247→210680 | 477 | HEAT repeat (*Diaphorina citri*) | XM_008472518.1 | 2.00E-25 |
| ORF22 | 220116→220664 | 182 | Dice1 (*Ixodes scapylaris*) | XM_00243505.1 | 5.6 |
| ORF23 | 252897→255455 | 264 | Ankyrin-3-like  (*Microplitis demolitor*) | EF710639.1 | 6.00E-16 |
| ORF24 | 256864→257831 | 112 | Small ribosomal RNA (Uncultured bacterium) | FJ354252.1 | 2.6 |
| ORF25 | 262898←263926 | 342 | DNA polymerase  (*Microplitis demolitor*) | XM_008555013.1 | 1.00E-05 |
| ORF26 | 264236→264551 | 93 | G3 protein  ( *Trichomonas vaginalis*) | XM_001299242.1 | 2.5 |
| ORF27 | 267510→268313 | 267 | Chromosome 2R  (*Drosophila melanogaster*) | AE013599.5 | 2E.67 |
| ORF28 | 272506→272967 | 153 | Retrotransposon TROMB (*Drosophila melanogaster*) | AJ277432.1 | 0.16 |
| ORF29 | 274649←275598 | 645 | Furin-like protease 2  (*Plutella xylostella*) | XM_011555912.1 | 2.00E-11 |
| ORF30 | 275023←275253 | 416 | Vankyrin 3 (*Microplitis demolitor* bracovirus) | XM_008555164.1 | 3.00E-41 |
| ORF31 | 275777→279515 | 74 | Integrase core protein (*Cotesia congregata* bracovirus) | KP706800.1 | 0.14 |
| ORF32 | 276724←277347 | 360 | E3 ubiquitin-protein ligase E3D (*Microplitis demolitor*) | XM_008545734.1 | 6.00E-16 |
| ORF33 | 277981←278412 | 296 | ATPase  (*Cotesia congregata* bracovirus) | AJ632329.1 | 5E-45 |
| ORF34 | 288642→290243 | 494 | PTP-α  (*Cotesia sesamiae* bracovirus) | CP007235.1 | 1.00E-94 |
| ORF35 | 288739←289047 | 102 | TSP13 (*Microplitis demolitor*) | JX399877.1 | 1E-08 |
| ORF36 | 289047←290243 | 117 | PTP-α (*Cotesia congregata* bracovirus) | DQ839630.1 | 2.00E-35 |
| ORF37 | 290837→294481 | 542 | Synapsin (Atta cephalotes) | XM_012208289.1 | 3E-41 |
| ORF38 | 291453→ 291890 | 37 | ATPase (*Cotesia congregata* bracovirus) | AJ632317.1 | 2.30E+00 |
| ORF39 | 299424→299605 | 182 | PTP-H (*Cotesia congregata* bracovirus) | AJ640092.1 | 6E-111 |
| ORF40 | 306544←31737 | 1394 | Integrase core protein (*Trichinella spiralis*) | XM_003375197.1 | 1.00E-52 |
| ORF41 | 323572←325314 | 580 | Transposable element (*Megachile rotundata*) | XM_012294349.1 | 2.00E-44 |
| ORF42 | 329148→329708 | 188 | Serine/threonine protein kinase  (*Monomorium pharaonis*) | XM_012666479.1 | 3.00E-04 |
| ORF43 | 336807←337922 | 372 | β-Tubulin *(Plutella xylostella)* | XM_008565703.1 | 1.00E-86 |
| ORF44 | 342063→342821 | 252 | Chromosome 2R (*Drosophila melanogaster*) | AE013599.5 | 5.00E-03 |
| ORF45 | 395966←396535 | 189 | β-Tubulin  (*Bombyx mori*) | XM_012697798.1 | 7.00E-116 |
| ORF46 | 398010←398333 | 107 | β-Tubulin (*Ovis aries*) | GQ338157.1 | 7.00E-52 |
| ORF47 | 400412←400744 | 110 | HzNV orf124-like  (*Cotesia congregata*) | FN557481.1 | 1.00E-57 |
| ORF48 | 469832←470218 | 128 | ATPase (*Cotesia congregata* bracovirus) | XM_014441476.1 | 9.00E-46 |
| ORF49 | 471193←471648 | 151 | Polycomb protein PHO (*Ceratitis capitata*) | XR_190668.2 | 4.00E-20 |
| ORF50 | 501823→502593 | 256 | Zinc finger protein 628-like (*Copidosoma floridanum*) | XM_014350517.1 | 2.00E-67 |
| OFR51 | 561875-562258 | 127 | Pyruvate dehydrogenase kinase  (*Megachile rotundata*) | XM_011147643.1 | 8.00E-06 |
| ORF52 | 562311→562661 | 116 | Transcript variant X1  (*Apis mellifera*) | XM_391954.5 | 3.11E-30 |
| ORF53 | 563762→564730 | 322 | Transcript variant X2  (*Apis mellifera*) | XM_006558496.1 | 0.11 |
| ORF54 | 587638←588684 | 348 | Serine/threonine protein kinase  (*Microplitis demolitor*) | XM_014444487.1 | 0.00E+00 |
| ORF55 | 591910←592239 | 109 | Serine/threonine protein kinase 26 (*Microplitis demolitor*) | XM_008559860.2 | 3.00E-38 |
| ORF56 | 604842←605228 | 128 | Serine/threonine protein kinase 26 (*Fopius arisanus*) | XM_011312349.1 | 1.00E-06 |
| ORF57 | 623480←623800 | 106 | Heparan-sulfate 6-O-sulfotransferase (*Microplitis demolitor*) | XM_008557860.1 | 4.00E-29 |
